# Prevalence of Pipelining in the United States Orthopedic Surgery Residency Match

**DOI:** 10.7759/cureus.80303

**Published:** 2025-03-09

**Authors:** Chandler A Sparks, Edward V Contrada, Matthew J Kraeutler, Anthony J Scillia

**Affiliations:** 1 Orthopedic Surgery, Hackensack Meridian School of Medicine, Nutley, USA; 2 Orthopaedic Surgery and Rehabilitation, Texas Tech University Health Sciences Center, Lubbock, USA; 3 Sports Medicine/Orthopedics, Seton Hall University, Paterson, USA

**Keywords:** orthopedic match, orthopedic surgery, orthopedic surgery residency, pipelining, residency match

## Abstract

Background: Pipelining is the phenomenon whereby applicants from the same medical schools repeatedly match into the same residency programs. We sought to quantify the prevalence of pipelining in the United States (US) orthopedic surgery residency match and to compare these practices amongst geographic regions.

Methodology: Resident information was obtained from program webpages. New programs without five years of residents, programs that did not publicly report resident information, and programs with incomplete information were excluded. For the remaining programs, the pipelining ratio was calculated (pipelining ratio = no. of residents/no. of different medical schools represented over the study duration). We also recorded the proportion of each program’s residents that attended the single most represented medical school at each program during the study period and the number of years in which at least two residents from the same medical school matched into a program. Residency program geographic region and the number of medical schools within a 50-, 100-, and 200-mile radius of each program were also recorded.

Results: The median pipelining ratio amongst programs included (n = 159) was 1.5 (interquartile range (IQR) = 1.32-1.79; Range = 1-4.83). The pipelining ratio varied by geographic region (p<0.01), with programs in the Midwest (p = 0.04) and South (p = 0.04) having a higher pipelining ratio than programs in the Northeast. The proportion of each program composed of the most represented medical school varied by geographic region (p<0.01), with programs in the South having a higher proportion of their classes composed of residents from a single medical school than programs in the Northeast (p<0.01) and Western US (p = 0.03). The pipelining ratio and proportion of each program's residents from a single shared medical school showed no correlation or a very weak negative correlation with the number of medical schools within a 50-, 100-, and 200-mile radius of each program.

Conclusions: Most orthopedic residency programs fall within a range of fair to moderate pipelining, though it is more common in Southern US programs. These practices can limit opportunities for qualified applicants and should be monitored due to recent changes in pass/fail scoring of the US Medical Licensing Step 1 Exam and virtual interviews.

## Introduction

Orthopedic surgery is one of the most competitive United States (US) residency programs to match into, with a total of 1,425 applicants for 899 positions in the 2023 Match [1]. The competitiveness of this process is reflected in the large number of programs applied to by each applicant, which approximately doubled from an average of 41.1 programs per applicant in 2001 to 79.3 in 2018 [2]. The large number of programs applied to has encouraged an increased reliance on objective measures, such as test scores, alpha omega honor society membership status, and research productivity to evaluate applicants [2-4]. However, more personal evaluations of an applicant by residency programs (e.g., away rotations) also largely influence an applicant’s likelihood of matching at a particular program [5,6].

“Pipelining” is a term that recently has been used to describe the phenomenon by which applicants from certain orthopedic residency programs frequently match at the same sports medicine fellowship programs [7]. This term has also been used to describe a means of increasing diversity and cultural competency in the workforce [8-11]. Used in the latter context, “pipelining” is a positive term that is imperative for a positive work environment and quality patient care. This study focuses on pipelining in the former context, though applies it to residency program applicants rather than fellowship applicants. This practice can result from objective evaluations of applicants, whereby an applicant’s medical school is used to filter applicants due to the large number of applications received by each program. Conversely, it can also be a result from personal connections to the applicant and/or letter writer [7]. “Top Feeder Med Schools” to orthopedic residency programs is publicly available data through databases such as Doximity [12]. However, there has been little quantitative investigation into the occurrence of this for a successful match into orthopedic surgery.

An understanding of the evaluation factors for the orthopedic surgery match allows medical students to take an evidence-based approach to matching into it [2]. Furthermore, it is important to understand how residents are selected for its residency programs to recognize the potential for inequitable bias. The purpose of this investigation was to quantify the overall prevalence of pipelining in the US orthopedic surgery residency match and to make comparisons of these practices by US geographic regions.

## Materials and methods

Residency program selection and characteristics

Current US orthopedic residency programs were identified through the Electronic Residency Application Service® (ERAS®) 2024 Participating Specialties and Programs database [13]. Programs that did not have five years of residents at the time of the investigation (i.e., new programs) were not included. Current residents at each program and their respective medical schools were recorded from publicly available information on residency program webpages (official program websites and official program social media webpages). Programs that did not fully report current resident information were excluded from the present study. For the programs included, geographic region of the residency program was also recorded according to state, district, or territory where the program is located. Geographic regions by state/district/territory are summarized in Table 1. The number of medical schools within a 50-, 100-, and 200-mile radius of each program was also recorded. Medical schools without a graduating class (i.e., new schools) were not recorded. This study did not obtain data through intervention or interaction with individual human subjects and utilized data from publicly available databases; therefore, this study was deemed exempt from institutional review board's review.

**Table 1:** Program geographic regional categories by state, district, and territory

Region	State/District/Territory
Midwest	Ohio, Indiana, Michigan, Illinois, Missouri, Wisconsin, Minnesota, Iowa, Kansas, Nebraska, and North Dakota
Northeast	Massachusetts, Rhode Island, Connecticut, New Hampshire, Vermont, New York, Pennsylvania, New Jersey, Delaware, and Maryland
South	West Virginia, Virginia, District of Columbia, Kentucky, Tennessee, North Carolina, South Carolina, Georgia, Alabama, Mississippi, Arkansas, Louisiana, Puerto Rico, Florida, Texas, and Oklahoma
West	Colorado, New Mexico, Arizona, Washington, Oregon, Utah, Nevada, California, and Hawaii

Pipelining quantification

Program webpages were investigated during the month of July 2023. For each program, the number of current residents and the number of medical schools represented were obtained. These values were then used to calculate the pipelining ratio as described by Tanguilig et al., i.e., the ratio of the total number of residents over the duration of the study to the number of different medical schools represented within the residency program (pipelining ratio = no. residents/no. programs represented) [7]. The proportion of each program’s residents that attended the single most represented medical school at each program during the study period was also recorded (no. residents from most represented medical school/total no. residents). Lastly, the number of years out of the five years reported in which at least two residents from the same medical school matched into a program was also recorded. 

Statistical analysis

All statistical analysis was done in RStudio (Version 1.4.1106). Descriptive statistics are provided for all parameters recorded. Non-parametric tests were used due to the non-normal patterns of the data distribution. A Kruskal-Wallis test was used to make comparisons between the parameters recorded and geographic region. Where significance was found, a Dunn test with Benjamini-Hochberg adjustment was used for post hoc analysis. Continuous variables were analyzed using Spearman’s rank correlation coefficient. Statistical significance of the results from these tests was set apriori as those with p-values≤0.05.

## Results

Overall pipelining prevalence

**Figure 1:**
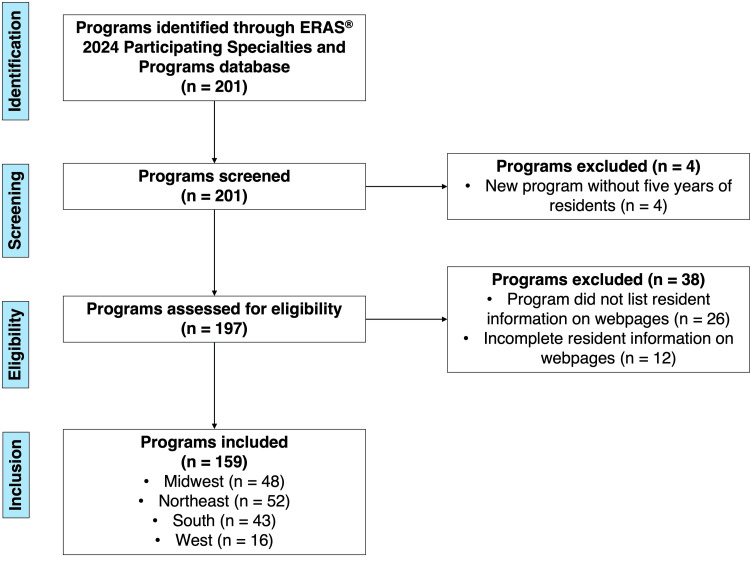
Search strategy for program selection

**Figure 2:**
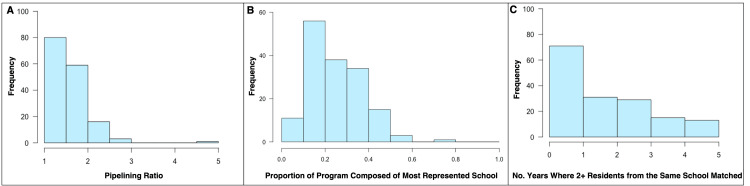
Histograms of pipelining parameters recorded. Parameters include pipelining ratio (A), the proportion of each program composed of the most represented medical school (B), and the number of years in which at least two residents from the same medical school matched at a program (C).

The ERAS® 2024 Participating Specialties and Programs database listed 201 programs, of which 42 were excluded due to failure to meet the above criteria (Figure 1). The median pipelining ratio amongst all programs included (n = 159) was 1.5 (interquartile range (IQR) = 1.32-1.79; Range = 1-4.83). The median proportion of each program’s residents that attended the single most represented medical school at each respective program during the study period was 0.24 (IQR = 0.16-0.33; Range = 0.04-0.72). The median number of years in which at least two residents from the same medical school matched at the same program was 2 (IQR = 1-3; Range = 0-5). Distributions of pipelining parameters are described in Figure 2.

Pipelining prevalence by geographic region

**Figure 3:**
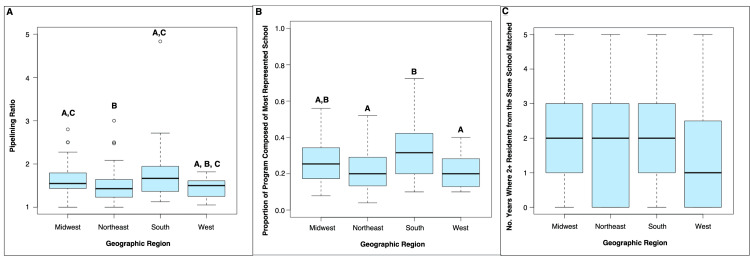
Boxplots of pipelining parameters by geographic region. Boxplots show pipelining ratio (A), the proportion of each program composed of the most represented medical school (B), and the number of years in which at least two residents from the same medical school matched at a program (C). There was a significant difference between pipelining ratio by geographic region (p<0.01) and the proportion of each program composed of the most represented medical school by geographic region (p<0.01). Groups sharing the same letters above bars are not significantly different (p>0.05) whereas groups with different letters are significantly different (p<0.05) per post hoc analysis.

There was a significant difference between pipelining ratio amongst programs of different geographic regions (p<0.01), as well as between the proportion of each program’s residents that attended the single most represented medical school at each respective program and geographic region (p<0.01). There was no significant difference between the number of years in which at least two residents from the same medical school matched at the same program and geographic region (p = 0.26). Programs in the Midwest had a significantly higher pipelining ratio than programs in the Northeast (median = 1.55 vs. 1.43; p = 0.04). Programs in the South also had a higher pipelining ratio than programs in the Northeast (median = 1.67 vs. 1.43; p = 0.04). Programs in the South had a higher proportion of their programs composed of residents from the single most represented medical school compared to programs in the Northeast (median = 0.32 vs. 0.20; p<0.01) as well as programs in the West (median = 0.32 vs. 0.20; p=0.03). These results are summarized in Figure 3. 

There was no correlation or a very weak negative correlation between the pipelining ratio and the number of medical schools within a 50-mile (ρ = -0.18; p = 0.03), 100-mile (ρ = -0.16; p = 0.05), and 200-mile radius (ρ = -0.14; p = 0.08) of each program. There was also no correlation or a very weak negative correlation between the proportion of each program’s residents from a single shared medical school and the number of medical schools within a 50-mile (ρ = -0.15; p = 0.06), 100-mile (ρ = -0.18; p = 0.03), and 200-mile (ρ = -0.15; p = 0.05) radius of each program.

## Discussion

The most important finding of this study is that the pipelining practices of orthopedic residency programs are variable. While some programs demonstrate little or no pipelining, others had a very high prevalence of it, with one program having nearly three-quarters of its program composed of residents from a single medical school. However, most programs fell within a range of fair to moderate pipelining. The practice of pipelining is most prevalent amongst programs in the Southern US, while Northeastern US programs have a lower prevalence. It should be noted, though, that programs in the same geographic region display a range of pipelining practices by residency programs. 

It is important for orthopedic surgery residency applicants to be aware of pipelining practices in order to take an evidence-based approach to matching into orthopedic surgery. Programs with a high degree of pipelining, while favorable to applicants who are within the network of schools that repeatedly match at a program, may not be ideal programs for applicants who are out of this network of schools. Applicants should be attentive to these practices when selecting programs for sub-internships, residency application, and sending preference signals. Applicants from schools without a home program may be especially disadvantaged by the practice of pipelining, given that applicants commonly match at their home programs due to increased physical exposure, with this becoming even more prevalent with the use of virtual interviews [14,15].

This analysis shows variable practices amongst programs, so applicants should consider the practices of programs on an individual program basis. Fortunately, this study did not identify a high prevalence of pipelining at most programs. However, we did identify that there are some programs that can be considered “pipelining hotspots” (n = 24 programs with a pipelining ratio≥2). Applicants may encounter a larger number of these programs in the Southern US (n = 11). The program directors (PDs) at these programs might place more emphasis on medical school attended, personal connection to the applicants, and/or personal connection to letter writers when evaluating applicants, though further investigation is needed to confirm this. It might be expected that programs with a larger number of nearby medical schools might have a lower prevalence of pipelining due to increased ability of students at these nearby schools to make personal connections and do sub-internships. However, this study found this association to be very weak.

PDs should also be aware of pipelining, especially within their own programs. Positive pipelining programs aimed at providing specialty exposure, mentorship, and interaction with faculty are vital for increasing diversity in the field of orthopedic surgery [8-11]. However, pipelining among orthopedic surgery residency programs, as described in the context of this study, may present a disadvantage for qualified orthopedic surgery residency applicants and have the opposite effect of the objectives set out by positive pipelining programs due to the applicant’s school taking priority over the remainder of the application [7]. Therefore, this practice should be kept minimal, given that qualified applicants who do not have the advantage of personal connections, “word-of-mouth” references, or attendance of a particular medical school may be disadvantaged and, thus, inequitable outcomes may occur. Tanguilig et al. have discussed that blinding of an applicant’s institution or blinding of the names of attendings submitting letters of recommendation are possible, though imperfect solutions to this issue and that concerted efforts to evaluate applicants without influence from personal connections is the most effective solution [7].

Recent changes to the residency application process may result in an increased prevalence of pipelining. First, the January 2022 change of pass/fail score reporting of the United States Medical Licensing Examination (USMLE) Step 1 will largely change the residency application review process. PDs may begin relying more on factors such as medical school reputation, applicant familiarity, and recommendation letters, which may promote pipelining [16]. Second, virtual interviews became a crucial component of the residency application process during COVID-19 in 2020. Now, in the post-COVID-19 era, many programs are choosing to continue using a virtual interview format and both the Association of American Medical Colleges and National Resident Matching Program recommend that residency and fellowship programs use a virtual interview format [17,18]. These recommendations are due to applicant preference and the reduced cost of virtual interviews, which may widen access and improve equity [17]. However, virtual interviews present the challenge of reduced personal connection between the applicant and program from both an applicant and PD perspective [19-21]. This raises the concern that programs may use an increased reliance on personal connections or “word-of-mouth” references [7,14,15,22]. Conversely, some recent changes, such as preference signaling, provide applicants a means of gaining attention from a program not due to personal connections or medical school attended and have resulted in a more even distribution of interviews [23]. Thus, trends in pipelining practices should be monitored and addressed in future studies, given these major changes to the residency application process.

There are limitations of this study to be noted. First, this study did not evaluate all orthopedic residency programs but was able to evaluate ~80% of programs with five years of residents. Second, this study does not evaluate the attitudes or priorities of PDs when evaluating applicants. This study does not provide information on temporal trends in pipelining practices, insights which might be valuable for understanding past-present and present-future trends in these practices as changes occur. This study also does not assess the degree to which these findings are driven from home program vs. non-home program matches. Lastly, the US match process is determined by both program rankings of applicants as well as applicant rankings of programs. The degree to which these findings are driven by program practices vs. applicant preferences were not ascertained in the present study. Future studies should investigate the attitudes and practices of PDs regarding pipelining, factors other than geographic region that might be associated with pipelining practices, and temporal trends in pipelining practices.

## Conclusions

Pipelining practices are variable amongst orthopedic surgery residency programs. Most programs fall within a range of fair to moderate pipelining, though it is more common in Southern US programs. These practices can disadvantage and limit the opportunities for qualified applicants. Trends in them should be monitored due to recent changes in pass/fail scoring of the US Medical Licensing Step 1 Exam and virtual interviews, which may promote pipelining practices.
